# The Prevalence and Characteristics of Anemia in Romanian Patients with Type 2 Diabetes: A Cross-Sectional Study

**DOI:** 10.3390/jcm13237306

**Published:** 2024-12-01

**Authors:** Laura Gaita, Bogdan Timar, Sandra Lazar, Simona Popescu, Oana Albai, Adina Braha, Romulus Timar

**Affiliations:** 1 Second Department of Internal Medicine, “Victor Babes” University of Medicine and Pharmacy, 300041 Timisoara, Romania; gaita.laura@umft.ro (L.G.); popescu.simona@umft.ro (S.P.); albai.oana@umft.ro (O.A.); braha.adina@umft.ro (A.B.); timar.romulus@umft.ro (R.T.); 2 “Pius Brînzeu” Emergency County Hospital, 300723 Timisoara, Romania; 3 Centre for Molecular Research in Nephrology and Vascular Disease, “Victor Babes” University of Medicine and Pharmacy, 300041 Timisoara, Romania; 4 First Department of Internal Medicine, “Victor Babes” University of Medicine and Pharmacy, 300041 Timisoara, Romania; sandra.lazar@umft.ro; 5 Department of Haematology, Emergency Municipal Hospital, 300254 Timisoara, Romania

**Keywords:** type 2 diabetes, anemia, chronic kidney disease, iron deficiency, interdisciplinary management

## Abstract

**Background/Objectives:** Anemia is a prevalent comorbidity of diabetes, and although various mechanisms have been shown to link these two conditions, their interaction has not been sufficiently explored. Our cross-sectional, non-interventional study aimed to evaluate the prevalence of anemia and its subtypes, as well as their interactions, in patients with type 2 diabetes (T2D). **Methods:** A total of 227 patients previously diagnosed with T2D were enrolled. These patients were assessed regarding their medical history and the evolution of their diabetes, and were screened for anemia. **Results:** Anemia was encountered in 32.6% of the 227 hospitalized patients previously diagnosed with T2D enrolled in this study. Its presence was associated with a higher prevalence of complications and comorbidities, such as chronic kidney disease (CKD), retinopathy, and atrial fibrillation. The most common types of anemia observed were those associated with CKD, other chronic conditions, and iron deficiency. A moderate, positive correlation (r = 0.307; *p* < 0.0001) has been observed between estimated glomerular filtration rate (eGFR) and hemoglobin, and a moderate, negative correlation has been observed between age and hemoglobin (r = −0.351; *p* < 0.0001), with the results also analyzed using multiple regression and ROC curve analysis. Additionally, a weak, positive, yet statistically significant correlation was observed between glycemic values and hemoglobin levels, which requires further research. **Conclusions:** Anemia is frequently encountered in patients with T2D, especially in those with increased age, decreased eGFR, and additional chronic degenerative complications or other comorbidities; thus, a systematic screening for an early diagnosis and interdisciplinary management is recommended for improved outcomes related to morbidity, mortality, and quality of life.

## 1. Introduction

Diabetes mellitus is currently considered one of the most significant global health emergencies, with 537 million diagnosed patients in 2021 and a prediction of 783 million patients in 2045. Its burden is caused by the disease itself, as well as its comorbidities and complications [1]. The already well-known chronic degenerative complications that contribute to increased mortality and morbidity include coronary heart disease, cerebrovascular disease, peripheral artery disease (the macrovascular complications), diabetic retinopathy, and diabetic kidney disease (the microvascular complications), and, occasionally included in the latter category, diabetic neuropathy [2]. Nevertheless, multiple other conditions have been associated with diabetes, such as metabolic dysfunction-associated steatohepatitis (MASH), obstructive sleep apnea (OSA), cancer, affective disorders, or cognitive impairment, all of which are considered emerging complications and are beginning to be addressed in various guidelines and position statements [3,4,5].

One of the comorbidities frequently discovered, yet often overlooked in patients with diabetes, is anemia, with a prevalence of 25–40%, depending on the country and other characteristics of the included subjects [6,7,8,9]. This condition, defined by a low level of hemoglobin (Hb) concentrations, hematocrit, or red blood cell (RBC) numbers, has been shown to affect about a quarter of the general population across the globe, with higher figures reported in low- and middle-income countries. It is particularly prevalent among vulnerable groups, including children and women of reproductive age, and even more so in pregnant and postpartum women [10,11]. Anemias are associated with higher mortality and morbidity rates across all age groups and genders, contributing to a multitude of vulnerabilities. These include poor birth outcomes, impaired development, and increased susceptibility to infections in children; fatigue, lower productivity, and challenges in everyday activities in adults; and poor prognoses in hospitalized older adults [12,13]. Iron deficiency is the most common cause of anemia in the general population and the most prevalent nutritional deficiency worldwide, and has been linked to poor dietary intake, gastrointestinal diseases, or gynecological conditions. Additionally, anemias have been investigated, prevented, or treated in the context of infectious diseases and chronic kidney disease but are rarely mentioned in recommendations for other non-communicable conditions. However, iron deficiency has recently become a key component in the management of chronic heart failure, leading to new interdisciplinary approaches [14,15,16,17]. 

One of these interrelations, namely the one between anemia and diabetes, is insufficiently explored and mentioned throughout the guidelines. However, a low level of Hb has been independently associated with an increased risk of micro- and macrovascular complications and even with an increased risk of mortality in this category of patients [18]. Various mechanisms have been shown to link the two conditions, including chronic kidney disease (CKD), the main cause of anemia in patients with diabetes, autoimmune gastritis, and pernicious anemia in patients with type 1 diabetes. Additionally, multiple studies have described potential associations between increased iron intake during pregnancy and the risk of gestational diabetes mellitus. Emerging but controversial evidence also suggests a possible impact of iron deficiency on worsening blood glucose control and, conversely, the impact of poor glycemic control on the development of anemias [19,20,21]. The links between anemia and diabetes also include the difficulty in using hemoglobin A1c/glycated hemoglobin (HbA1c) values in cases with low Hb or erythrocyte indices, both major confounders in correctly interpreting this key parameter in diagnosing diabetes and assessing glycemic control [22]. Furthermore, various antihyperglycemic agents have been proven to impact the development or prevention of anemia—the long-term use of metformin contributes to a vitamin B12 deficiency and low levels of Hb, while, in contrast, the use of sodium-glucose cotransporter-2 inhibitors (SLGT2i) has been associated with beneficial effects such as an increased hematocrit and erythropoiesis [23,24,25,26,27,28]. Additionally, the early diagnosis and treatment of anemia have been associated with a decreased risk of mortality, morbidity, and healthcare utilization, and with an improved quality of life—essential aspects in the holistic management of patients with type 2 diabetes (T2D) [29,30]. 

Nevertheless, although diabetes and anemia are very prevalent in the general population and a low level of Hb is frequently encountered in patients with diabetes, with an intricate interrelation, as previously mentioned, few studies have aimed to assess the prevalence of anemia in diabetes and the characteristics of these patients, especially in Central and Eastern European countries, such as Romania. With these premises, our study aimed to assess the prevalence of anemia, in general, as well as the different types of anemia, specifically in patients with T2D. It also aimed to explore the associations between anemia and various metabolic parameters, with the intention of contributing to an improved quality of interdisciplinary care of these patients. 

## 2. Materials and Methods

### 2.1. Study Design and Patients

In this cross-sectional, non-interventional study, we enrolled 227 patients already diagnosed with T2D who were hospitalized in the Department of Diabetes, Nutrition and Metabolic Diseases of the “Pius Brînzeu” Emergency County Hospital in Timișoara, Romania, between March and July 2024. The study protocol, design, and also the informed consent form were previously approved by the Ethics Committee of the “Pius Brînzeu” Emergency County Hospital in Timișoara (no. 488/30 September 2024), which is the only requirement for non-interventional research, according to local protocols. The study was conducted according to the principles stated in the Declaration of Helsinki, and all patients provided written informed consent before any study activities. The inclusion criteria were age > 18 years and a prior diagnosis of T2D. The exclusion criteria included ongoing pregnancy, inability to provide informed consent or an accurate anamnestic medical history, severe neurological or psychiatric disorders, or suspicion of other types of diabetes except for T2D. The report of this observational study was prepared according to the STROBE (Strengthening the reporting of observational studies in epidemiology) statement checklist (Supplementary File S1) [31]. 

### 2.2. Clinical, Anthropometric, and Laboratory Data

Data regarding the patient’s gender, age, duration of diabetes, weight, body mass index (BMI), and history of blood loss were collected from the patient’s medical records, while the interdisciplinary diagnoses of hypertension, coronary artery disease, peripheral artery disease, stroke, heart failure, atrial fibrillation, and MASH were established through interdisciplinary consults of cardiology and gastroenterology. BMI was calculated as weight (kg)/height^2^ (m). Fasting plasma glucose (FPG) levels, postprandial glucose (PPG) levels, HbA1c, hemoglobin (Hb), red blood cell (RBC) count, hematocrit, mean corpuscular volume (MCV), mean corpuscular hemoglobin concentration (MCHC), mean corpuscular hemoglobin (MCH), lipid profile, liver enzymes, bilirubin, serum creatinine, serum uric acid, serum iron, ferritin, serum vitamin B12, and folate levels were measured after at least 12 h of fasting, using standardized methods in the “Pius Brînzeu” Emergency County Hospital laboratory. Additionally, the urinary albumin/creatinine ratio was analyzed in the same laboratory using standardized methods from a spot urine sample. 

Regarding the diagnosis of microvascular complications of diabetes, the presence of retinopathy was established through a fundoscopic examination performed by a trained ophthalmologist, the presence and severity of CKD were established according to the Kidney Disease: Improving Global Outcomes (KDIGO) guidelines, and the presence of diabetic neuropathy was assessed using the 10 g monofilament test, temperature sensation test, pinprick test, and vibration perception test with a 128 Hz tuning fork [32,33]. Regarding the diagnosis of macrovascular complications of diabetes and other CV diseases, the presence of peripheral artery disease was established using the ankle-brachial index, whereas the presence of coronary artery disease or heart failure was established according to the European Society of Cardiology (ESC) guidelines [34,35]. Additionally, systolic and diastolic blood pressure measurements were performed following the recommendations of the ESC, and hypertension was defined as an SBP ≥ 140 mmHg and/or DBP ≥ 90 mmHg, a self-reported history of physician-diagnosed hypertension, or the use of antihypertensive agents, while the eGFR was calculated using the CKD-EPI creatinine equation (2021) [36].

### 2.3. Anaemia Assessment

The presence of anemia was defined as a Hb lower than 13.5 g/dL in men and 12 g/dL in women. Anemia was evaluated using the MCV, MCH, MCHC, serum iron level, ferritin level, serum vitamin B12 level, serum folate level, and data regarding a history of blood loss or other relevant comorbidities. Microcytic anemia was defined as an MCV lower than 80 fL, macrocytic anemia as an MCV higher than 100 fL, and normocytic anemia as an MCV between 80 and 100 fL. Additionally, hypochromic anemia was defined as either an MCH lower than 27 pg or an MCHC lower than 32 g/dL, while normochromic anemia was defined as an MCH between 27 and 32 pg and an MCHC between 32 and 26 g/dL [10,37,38].

Iron deficiency anemia was diagnosed in patients with microcytic hypochromic anemia and a serum iron level < 33 μg/dL or a serum ferritin level < 30 ng/mL. In the presence of macrocytic anemia, the serum levels of vitamin B12 (considered low below 197 pg/mL) and folate (considered low below 4.6 ng/mL) were measured. Anemia in CKD was defined as the presence of CKD itself, while also being an exclusion diagnosis, with other causes being evaluated in the presence of iron deficiency or significant comorbidities. The presence of CKD was established according to the KDIGO 2024 guideline criteria, namely an eGFR lower than 60 mL/min/1.73 m^2^ or an ACR higher than 30 mg/g, maintained over the course of at least 3 months. Similarly, anemia associated with other chronic diseases was diagnosed in the presence of cancer, heart failure, inflammatory bowel disease, or rheumatic diseases, after excluding other causes. Lastly, the presence of posthemorrhagic anemia was established considering patients’ history of blood loss and, if still suspected, after an additional gastroenterological consultation [32,39,40].

### 2.4. Statistical Analysis

In order to perform the statistical analysis, MedCalc^®^ Statistical Software version 20.210 was used (MedCalc Software Ltd., Ostend, Belgium; https://www.medcalc.org; 2022, accessed on 5 October 2024). Numerical variables with a Gaussian distribution are represented as mean ± standard deviation, while numerical variables with a non-parametric distribution are represented as median and interquartile range. Nominal variables are presented as percentages of the subgroup total or as frequencies. We applied the Shapiro–Wilk method to assess the normality of the distribution of the numerical variables. A *p*-value lower than 0.05 obtained in this assessment corresponds to non-parametric distributions.

In order to assess the differences between the indicators of the central tendency, we used the Mann–Whitney U test to compare two medians and the unpaired Student’s *t*-test to compare the arithmetic means of the parametric variables between two groups. The ANOVA test was used to study the variation in the mean values between at least two groups. We also used the chi-square test (for comparison of two proportions) to test the statistical significance of the differences between these proportions.

We used correlation coefficients in order to assess the strength and direction of the associations between numerical variables, and calculated the coefficient of determination (R^2^) in the case of bivariate regressions. For evaluation of the predictive power of anemia, we performed “Receiver-Operating Characteristics” analyses, with performance described in terms of sensitivity and specificity. The predictor’s optimal threshold value was considered equal to the Youden index. We have also compared the area under the ROC curves of the model created with that of a non-discriminant model in order to analyze the statistical significance of its predictive capacity. Additionally, multivariate logistic regression models were built to evaluate the impact of age and eGFR on the presence of anemia. 

The sample size was calculated before enrollment, taking into account literature data in order to provide a confidence level of 95% and a statistical power higher than 80%. Moreover, this study considered a *p*-value lower than 0.05 as the statistical significance threshold.

## 3. Results

After considering the inclusion and exclusion criteria, the study group consisted of 227 patients with T2D, of which 52.9% (120) were women, with a median age of 66 [57.3; 73] years, a median duration of diabetes of 10 [4; 16] years, and a median HbA1c of 8.1% [6.9; 9.8]. A detailed overview of the characteristics of the studied sample, compared by gender, is presented in Table 1. 

Anemia was diagnosed in 74 of the 227 patients in the studied sample, leading to a prevalence of 32.6% (CI 95%: 25.6–40.9%). Regarding the types of anemia, the most frequently encountered was anemia associated with CKD (in 29 patients with a prevalence of 12.8% in the studied sample), followed by anemia associated with other chronic conditions, such as cancer, heart failure, inflammatory bowel disease, or rheumatic diseases (in 23 patients with a prevalence of 10.1% in the studied sample), and then by iron deficiency anemia (in 17 patients with a prevalence of 7.5% in the studied sample). The distribution of anemia types is shown in Table 2. 

Regarding complications and comorbidities, the analysis of the studied patients revealed a statistically significant difference between those with and without anemia, with a higher prevalence of atrial fibrillation (22.9% vs. 9.1%, *p* = 0.004), chronic kidney disease (64.9% vs. 43.1%, *p* = 0.0022), and retinopathy (52.7% vs. 28.8%, *p* = 0.0005), namely, cardiovascular and microvascular disease, in patients with T2D and anemia. This comparison, based on the presence of anemia, is presented in Table 3. 

A moderate, positive, statistically significant correlation (r = 0.307; *p* < 0.0001) has been observed between eGFR and Hb, suggesting that anemia is more likely to develop in patients with decreased kidney function. This tendency is emphasized by another statistically significant correlation, this time a weak, negative one, between serum creatinine and Hb (r = −0.232; *p* = 0.0004), while ACR and Hb have not been observed to correlate significantly. Additionally, a moderate, negative, statistically significant correlation was observed between age and Hb level (r = −0.351; *p* < 0.0001), suggesting an increased prevalence of anemia in the older population. Another positive but weak statistically significant correlation was observed between weight or BMI and Hb (r = 0.254; *p* = 0.0001 for weight and r = 0.152; *p* = 0.021 for BMI), suggesting that anemia is associated with a lower weight, also represented by a lower BMI. Finally, a weak, positive, yet statistically significant correlation was observed between FPG or PPG and Hb (r = 0.179; *p* = 0.006 for FPG and r = 0.141; *p* = 0.034 for PPG), suggesting that T2D patients with anemia could have lower glycemic values, which was not confirmed by a significant correlation between HbA1c and Hb. The correlations between these parameters, additional characteristics, and Hb values are presented in Table 4. 

To evaluate the extent to which these parameters influence Hb (the dependent variable), taking into consideration their interaction, we performed a multivariate analysis that included all the parameters as the initial step, followed by successive eliminations and retesting in a backward-stepwise approach. The subtraction criterion of the predictor was a *p*-value > 0.1, whereas the addition criterion was a *p*-value < 0.05. The most statistically significant model, presented in Table 5, included age and eGFR, with an R^2^-adjusted value of 0.136 and a multiple correlation coefficient of 0.3798 (*p* = 0.0001). 

**Figure 1:**
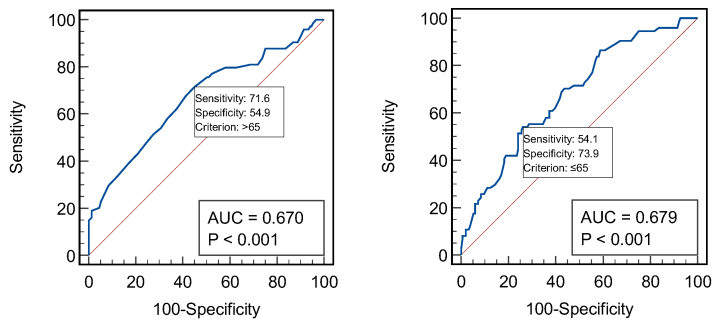
Graphical representation of the ROC curve analysis of anemia for age (**left**) and eGFR (**right**).

We performed an ROC curve analysis to assess the association of anemia with age and eGFR, and both parameters were shown to correlate significantly with Hb and were included in the previous multiple regression analysis. According to the ROC curves, an age > 65 years or an eGFR ≤ 65 mL/min/1.73 m^2^ represent statistically significant predictive factors for anemia, with a sensitivity and specificity of 71.6 and 54.9, respectively, for age (AUROC = 0.670, *p* < 0.001) and a sensitivity and specificity of 54.1 and 73.9, respectively, for eGFR (AUROC = 0.679, *p* < 0.001) (Figure 1). 

## 4. Discussion

### 4.1. Findings and Interpretation

This study analyzed the prevalence of anemia in patients with T2D and found a percentage of 32.6% in the selected sample, which is higher than the prevalence in the general population, reported by various studies to be approximately 25% [11,41]. Moreover, the value is similar to that reported in the population of patients with diabetes, which varies between 25 and 40%, depending on the country and other characteristics of the included subjects [7,8,9,42,43]. 

Regarding the type of anemia, this study has shown that the main cause in the sample of patients with T2D was anemia associated with CKD, followed by anemia associated with other chronic conditions such as cancer, heart failure, inflammatory bowel disease, rheumatic diseases, and iron deficiency anemia. These were followed, at a much lower prevalence, by posthemorrhagic and macrocytic or vitamin deficiency anemia, such as those caused by vitamin B12 or folate deficiency. It is noteworthy that, although iron deficiency is the most common cause of anemia in the general population, CKD is the main cause in patients with diabetes, although, in many cases, the mechanisms that lead to anemia remain unknown [6,15]. Nevertheless, diabetes is the leading cause of CKD, and even more so, patients with T2D in this study were admitted to the Department of Diabetes, suggesting a higher prevalence of chronic complications and increased severity of the condition. This led to a higher probability of encountering CKD, with a prevalence of 50.2% in this study compared to the 20–50% reported in various other studies [44,45]. 

Additionally, the results indicate a statistically significant correlation between lower Hb levels in T2D patients and increased age, worse kidney function as reflected by a lower eGFR, and lower weight and BMI. Furthermore, although a correlation between lower Hb levels and a higher urinary albumin/creatinine ratio (UACR) was suggested, it was not statistically significant. While the increase in the prevalence of anemia with aging is well known and is consistent among various studies, the results regarding lower weight and BMI are conflicting, indicating either a positive or a negative correlation between Hb, weight, and BMI [9,46,47]. Nevertheless, anemias are frequently caused by a nutritional deficiency of either iron or vitamins, including CKD-related anemia, which is also partly caused by nutritional deficiencies in addition to a functional erythropoietin deficiency or inflammation [48,49]. Moreover, the association between anemia and lower weight or BMI could be related to other chronic diseases usually associated with unintentional weight loss, especially in severe cases and chronic conditions frequently encountered in the study sample [50,51,52]. Additionally, the association between anemia and impaired kidney function has been highlighted by multiple studies, with a lower Hb encountered in patients with lower eGFR, anemia being considered a strong predictor of mortality in patients with T2D and CKD [42,53,54]. 

With regard to comorbidities such as atrial fibrillation and microvascular complications (diabetic retinopathy and, as expected, CKD), this study has also shown that in patients with T2D and anemia, their prevalence is increased. These results are in substantial concordance with other studies, which have shown that anemia in diabetes patients is associated with an increased risk of developing CVD and microvascular complications, and that its treatment can lead to improved patient-related outcomes. This interaction still needs further investigation regarding causality, especially since anemia could be caused by DKD, with incertitude regarding the cause-effect relation between anemia and retinopathy, while the bidirectional interaction between anemia and atrial fibrillation, in patients with and without diabetes, is already well known [18,55,56,57,58,59]. 

This study also aimed to analyze the interaction between glycemic control and anemia, a subject of controversy as a result of the multiple mechanisms by which iron deficiency could contribute to increased glycemic values by affecting glucose tolerance, insulin synthesis, and glucose metabolism, as described by various studies. Moreover, it appears that the presence of diabetes could contribute to iron deficiency due to nutritional imbalances with insufficient intake, chronic inflammation, and malabsorption caused by diabetic gastroparesis and enteropathy, in addition to changes in iron metabolism itself [60,61,62,63]. Nevertheless, the assessment of the effect of glycemic control on Hb levels, and the reverse process, should include measurements of blood glucose levels. Potential confounding results may arise when considering HbA1c, expressed either as % or mmol/mol, as a consequence of the inaccuracy of this parameter in patients with anemia [64]. In this study, a statistically significant positive correlation was observed between both fasting and postprandial plasma glucose levels and the level of Hb, without any correlation between the levels of HbA1c and Hb; however, further research is needed to characterize the interaction between these parameters.

### 4.2. Strengths and Limitations of the Study

One of the strengths of this research is the consecutive enrollment of patients, which led to obtaining a cohort that included individuals with T2D with and without anemia with heterogeneous characteristics such as age, sex, duration of diabetes, weight, glycemic and lipid control, and complication profile, reflecting the diversity of patients encountered in daily clinical practice. Moreover, the sample size is another strength of this research, providing sufficient statistical power and thus allowing the inference of the results for the population of patients with T2D. 

The main limitation of this study is its cross-sectional design, which does not allow a time-dependent analysis of the interaction between anemia, comorbidities, and complications of diabetes, as well as the interaction between glycemic control and anemia. Nevertheless, this aspect does not interfere with the main aims of the study, yet it could represent a starting point for future research. Moreover, the selection of the study sample from patients admitted to the Department of Diabetes, Nutrition, and Metabolic Diseases at the “Pius Brînzeu” Emergency County Hospital in Timișoara led to a population of patients with diabetes with a higher prevalence of complications and comorbidities, poorer glycemic control, and more severe disease progression. Lastly, details regarding the antihyperglycemic treatment of patients, measurements of erythropoietin, and, in some cases, ferritin have not been available in this study. These parameters could be included in future research for a more detailed analysis of the characteristics and causes of anemia. 

### 4.3. Relevance of the Findings

The results of this study highlight the importance of actively screening for anemia in patients with diabetes since the two conditions are frequently associated in real-life practice, with a negative prognosis of these patients caused by an increased risk of developing chronic degenerative complications or other comorbidities. This research highlights the extent to which patients with T2D also present with a low level of Hb, especially in the context of advanced age, impaired kidney function, or additional chronic conditions, emphasizing the need for additional vigilance and early, systematic screening when managing such cases. Additionally, these results could contribute to changes in the multifactorial approach of patients with T2D, transitioning from a cardio-reno-metabolic point of view to a holistic intervention that could also screen for anemia, investigate patients in detail when needed, and choose personalized treatments according to new guidelines and standards of care. This would involve interdisciplinary collaboration with hematology, subsequently decreasing risks of mortality and morbidity and improving quality of life. 

### 4.4. Future Perspectives

As previously mentioned, a starting point for future research is represented by the transformation of the design of this study from a cross-sectional to a prospective model, which could assess the time-dependent interaction between glycemic levels and the presence of anemia or the effect of low levels of Hb and the presence of chronic degenerative complications of T2D or its comorbidities. Furthermore, future studies could include additional parameters that could contribute to an in-depth characterization of anemia (such as erythropoietin or reticulocyte count) or additional information regarding chronic conditions that could lower Hb values. Another piece of information that should be included in an upcoming prospective study is the antihyperglycemic treatment of each patient with T2D since some agents, such as metformin or SGLT2i, have been proven to influence anemia. Nevertheless, it becomes evident that future research, performed using an interdisciplinary approach, is needed to obtain more knowledge about the association between anemias and diabetes and the practical steps that could be implemented in daily clinical practice to reduce mortality, morbidity, and increase the quality of life of these patients. 

## 5. Conclusions

The prevalence of anemia in patients with T2D is approximately 32.6%, which is higher than that of the general population, with a predominance of anemia associated with CKD, other chronic conditions, or iron deficiency anemia. Moreover, the presence of anemia in this population of patients is significantly correlated with increased age and impaired kidney function; however, the interaction between anemia and glycemic control or weight should be further investigated. In this context, individuals with T2D, especially in the context of advanced age, decreased eGFR, or additional chronic degenerative complications or other comorbidities, should be screened early and systematically for anemia. If anemia is detected, patients should be referred to a hematological evaluation for individualized, multifactorial, and interdisciplinary management that could contribute to improved outcomes related to morbidity, mortality, and quality of life. 

## Figures and Tables

**Table 1:** Comparison of the general characteristics of the studied sample by gender.

Parameter	Overall	Men	Women	*p*-Value *
% (number)	100% (227)	47.1% (107)	52.9% (120)	-
Age (years) ^a^	66 [57.3; 73]	65	68	0.012 **
T2D duration (years) ^a^	10 [4; 16]	10	10	0.309
Weight (kg) ^a^	84 [71; 98]	91	80	<0.0001 **
BMI (kg/m^2^) ^a^	30 [26; 34]	29	31	0.191
SBP (mmHg) ^b^	132.7 ± 19.4	128.9 ± 18.2	135.9 ± 19.8	0.006 **
DBP (mmHg) ^a^	78 [70; 85]	78	78	0.712
FPG (mg/dL) ^a^	130 [115;155]	130	130	0.912
PPG (mg/dL) ^a^	154 [130; 188]	152	155	0.682
HbA1c (%) ^a^	8.1 [6.9; 9.8]	8.3	8.1	0.538
Hb (g/dL) ^b^	13.5 ± 2.1	14 ± 2.1	13.1 ± 2	0.001 **
RBC count (mil/mm^3^) ^b^	4.59 ± 0.74	4.72 ± 0.8	4.47 ± 0.63	0.009 **
Ht (%) ^b^	39.7 ± 6.3	41 ± 6.6	38.4 ± 5.7	0.001 **
MCV (fL) ^a^	87 [83.7; 90]	88.3	86	0.003 **
MCH (pg) ^a^	29.7 [28.4; 31.1]	29.9	29.6	0.334
MCHC (g/dL) ^a^	34 [33.2; 34.7]	33.8	34.1	0.622
TC (mg/dL) ^a^	152 [126.3; 196.5]	151	152	0.819
LDLc (mg/dL) ^a^	85 [65; 119.8]	89	83	0.717
HDLc (mg/dL) ^a^	42 [35; 49]	40	43	0.003 **
TG (mg/dL) ^a^	141 [105; 193.8]	146	139	0.613
AST (U/L) ^a^	26 [21; 33.8]	25	26	0.891
ALT (U/L) ^a^	25 [19; 36]	29	24	0.308
TB (mg/dL) ^a^	0.6 [0.46; 0.87]	0.6	0.5	0.008 **
DB (mg/dL) ^a^	0.2 [0.13; 0.3]	0.2	0.2	0.134
Creatinine (mg/dL) ^a^	0.9 [0.7; 1.2]	1	0.8	0.0002 **
eGFR (mL/min/1.73 m^2^) ^a^	81 [54.3; 98.8]	81	79.5	0.235
ACR (mg/g) ^a^	24 [13.3; 71.5]	24	24	0.913
UA (mg/dL) ^a^	5.4 [4.4; 6.4]	5.9	5.1	0.032 **

^a^ Continuous variables (with non-Gaussian distribution) are indicated by their median [interquartile range]. Mann–Whitney test. ^b^ Continuous variables (with Gaussian distribution) are indicated as mean ± standard deviation. * *p*-value for comparison of variables according to gender; ** *p*-value < 0.05 (statistical significance). T2D—type 2 diabetes; BMI—body mass index; SBP—systolic blood pressure; DBP—diastolic blood pressure; FPG—fasting plasma glucose; PPG—postprandial glucose; HbA1c— hemoglobin A1c; Hb—hemoglobin; RBC—red blood cells; Ht—hematocrit; MCV—mean corpuscular volume; MCH—mean corpuscular hemoglobin; MCHC—corpuscular hemoglobin concentration; TC—total cholesterol; LDLc—low-density lipoprotein cholesterol; HDLc—high-density lipoprotein cholesterol; TG—triglycerides; AST—aspartate transaminase; ALT—alanine transaminase; TB—total bilirubin; DB—direct bilirubin; eGFR—estimated glomerular filtration rate; ACR—albumin/creatinine ratio; UA—uric acid.

**Table 2:** Types of anemia found in the studied sample of patients with T2D.

Type of Anemia	% (Number)
Iron deficiency anemia	7.5 (17)
Macrocytic anemia	0.9 (2)
Anemia associated with CKD	12.8 (29)
Anemia associated with other chronic conditions	10.1 (23)
Posthemorrhagic anemia	1.3 (3)

CKD—chronic kidney disease.

**Table 3:** Comparison of the comorbidities and chronic degenerative complications of the studied sample according to the presence of anemia.

Comorbidity or Complication	Total % (Number)	With Anemia % (Number)	Without Anemia % (Number)	*p*-Value *
Hypertension	91.6 (208)	90.5 (67)	92.1 (141)	0.680
Coronary artery disease	52.4 (119)	52.7 (39)	52.3 (80)	0.953
Cerebrovascular disease	20.7 (47)	31.1 (23)	15.7 (24)	0.074
Peripheral artery disease	17.6 (40)	24.3 (18)	14.4 (22)	0.065
Heart failure	35.2 (80)	29.7 (22)	22.2 (34)	0.079
Atrial fibrillation	13.7 (31)	22.9 (17)	9.1 (14)	0.004 **
MASH	50.7 (115)	32.4 (24)	59.4 (91)	0.0001 **
Chronic kidney disease	50.2 (114)	64.9 (48)	43.1 (66)	0.0022 **
Retinopathy	36.6 (83)	52.7 (39)	28.8 (44)	0.0005 **
Neuropathy	86.8 (197)	87.8 (65)	86.3 (132)	0.745

* Chi-squared test. ** *p*-value < 0.05 (statistical significance). MASH—metabolic dysfunction-associated steatohepatitis.

**Table 4:** Correlation between patients’ characteristics and Hb values.

Parameter	Hb *	*p*-Value
Age	−0.351	<0.0001 **
T2D duration	−0.121	0.068
Weight	0.254	0.0001 **
BMI	0.152	0.021 **
HbA1c	0.051	0.4453
FPG	0.179	0.006 **
PPG	0.141	0.034 **
Creatinine	−0.232	0.0004 **
eGFR	0.307	<0.0001 **
ACR	−0.049	0.4599

* Pearson correlation coefficient. ** *p*-value < 0.05 (statistical significance). Hb—hemoglobin; T2D—type 2 diabetes; BMI—body mass index; HbA1c—hemoglobin A1c; FPG—fasting plasma glucose; PPG—postprandial glucose; eGFR—estimated glomerular filtration rate; ACR—albumin/creatinine ratio.

**Table 5:** Multiple regression analysis between Hb and the studied parameters.

Independent Variables	Coefficient	Standard Error	t	*p*	r_partial_	r_semipartial_
(Constant)	15.6433					
Age	−0.04881	0.01352	−3.609	0.0004	−0.2344	0.2231
eGFR	0.01360	0.005759	2.362	0.0190	0.1559	0.1460

eGFR—glomerular filtration rate.

## Data Availability

The data presented in this study are available on request from the corresponding author. The data are not publicly available due to local privacy and data protection regulations.

## Supplementary Materials

The following supporting information can be downloaded at https://www.mdpi.com/article/10.3390/jcm13237306/s1, Supplementary File S1: STROBE Statement—Checklist of items that should be included in reports of cross-sectional studies.
